# Metformin suppresses NFE2L1 pathway activation to inhibit gap junction beta protein expression in NSCLC

**DOI:** 10.1002/cam4.7021

**Published:** 2024-04-01

**Authors:** Shuo Yu, Hui Ren, Tingting Liu, Xiaoyan Han, Hui Guo, Qian Ning, Yang Li, Hong Zhou, Mingwei Chen, Tinghua Hu

**Affiliations:** ^1^ Department of Respiratory and Critical Care Medicine The First Affiliated Hospital of Xi'an Jiaotong University Xi'an Shaanxi China; ^2^ Department of General Surgery The Second Affiliated Hospital of Xi'an Jiaotong University Xi'an Shaanxi China; ^3^ Department of General Surgery Weifang People's Hospital Weifang Shandong China; ^4^ Department of Oncology The First Affiliated Hospital of Xi'an Jiaotong University Xi'an Shaanxi China

**Keywords:** gap junction proteins, metformin, NFE2L1, NSCLC, prognosis

## Abstract

**Objective:**

Non‐small‐cell lung cancer (NSCLC) is a deadly form of cancer that exhibits extensive intercellular communication which contributed to chemoradiotherapy resistance. Recent evidence suggests that arrange of key proteins are involved in lung cancer progression, including gap junction proteins (GJPs).

**Methods and Results:**

In this study, we examined the expression patterns of GJPs in NSCLC, uncovering that both gap junction protein, beta 2 (GJB2) and gap junction protein, beta 2 (GJB3) are increased in LUAD and LUSC. We observed a correlation between the upregulation of GJB2, GJB3 in clinical samples and a worse prognosis in patients with NSCLC. By examining the mechanics, we additionally discovered that nuclear factor erythroid‐2‐related factor 1 (NFE2L1) had the capability to enhance the expression of connexin26 and connexin 31 in the NSCLC cell line A549. In addition, the use of metformin was discovered to cause significant downregulation of gap junction protein, betas (GJBs) by limiting the presence of NFE2L1 in the cytoplasm.

**Conclusion:**

This emphasizes the potential of targeting GJBs as a viable treatment approach for NSCLC patients receiving metformin.

## 1 INTRODUCTION

Despite numerous advancements in its management in recent times, lung cancer continues to be a primary contributor to worldwide fatality rates.^1^ More than 85% of instances of lung cancer belong to NSCLC category, which frequently presents as lung adenocarcinoma (LUAD) and lung squamous cell carcinoma (LUSC) pathological phenotypes.^2^ Approximately one‐third of NSCLC patients experience limited success in reducing tumor size and extending survival through treatment efforts, primarily due to the rapid emergence of chemoresistance, leading to unfavorable treatment outcomes and a bleak patient prognosis.^3^ Moreover, numerous existing therapeutic approaches demonstrate restricted effectiveness and are linked to substantial adverse reactions, thereby constraining their usefulness in managing advanced NSCLC.^4^, ^5^, ^6^, ^7^ Nevertheless, further investigation is required to examine the mechanisms that control chemoresistance in NSCLC in order to improve the well‐being and longevity of individuals afflicted by this form of cancer.

The tumor microenvironment (TME) contains a diverse array of tumor and stromal cells, as well as arrange of cell‐ derived factors. Cancer treatment relies not only on targeting cancer cells but also on manipulating the TME to suppress tumor growth.^8^, ^9^ Cell–cell communication with the TME is markedly enhanced and closely linked to chemoresistance.^10^ Several different signaling mechanisms function within tumors, including gap junctional (GJ) signaling,^11^ chemical signaling,^12^ and direct contact between cell‐surface proteins.^13^, ^14^, ^15^

GJs are transmembrane complexes formed by a diverse array of connexin proteins that function to promote signaling between cells and to allow for ions and small molecules to pass between these cells. GJPs have increasingly been shown to play complex roles in arrange of pathogenic contexts, with dozens of studies that have linked the upregulation of connexins to better patient prognosis in the context ofprostate,^16^ pancreatic,^17^ breast,^18^ NSCLC,^19^ and colorectal cancer.^20^ On the other hand, there is evidence indicating that increased expression of connexin in oral squamous carcinoma (SCC),^21^ breast cancer,^22^ LUSC,^23^ and colorectal cancer^24^ can be linked to an unfavorable prognosis in certain instances. Although the involvement of gap junctional alpha (GJA) subfamily proteins in NSCLC has been extensively researched, the contribution of GJB proteins in this specific pathological condition is still unclear. To better clarify the role of GJPs in NSCLC, we treated these tumor cells with the hypoglycemic drug metformin. Metformin has previously been identified as a tumor suppressor that suppresses tumor cell stem‐like behavior, proliferation, metastasis, and angiogenesis is while enhancing chemo sensitivity, modulating tumor‐related factor production, and altering tumor‐related signaling activity.^25^, ^26^, ^27^

Herein, we identified GJBs as key genes that are upregulated in NSCLC cells and linked to patient prognosis. We further found that metformin could inhibit these proteins in vitro. At a mechanistic level, we identified the nuclear factor, NFE2L1 as a potential regulator of GJB transcription in this context. We also found that metformin (MET) treatment was sufficient to partially suppress NFE2L1‐mediated GJB expression. Together, these data highlight previously unknown mechanisms whereby metformin may be able to suppress tumor progression via inhibiting GJPs expression in NSCLC.

## 2 MATERIALS AND METHODS

### 2.1 Patients samples

The Ethics Committee of Clinical Research of Xi'an Jiaotong University approved this study, which aligned with the Declaration of Helsinki and local guidelines. All patients provided written informed consent. Patient samples were collected at the First Affiliated Hospital of Xi'an Jiaotong University from January 2011 to December 2014. Tumor and paired paracancerous tissue samples were collected from patients that had not undergone chemotherapy or radiotherapy, and all patients enrolled in this study had histopathological confirmed disease. All patient follow‐up was completed in 2020, and cases that were lost to follow‐up or died due to other causes were not included in this study. Liquid nitrogen was used to store all the samples.

### 2.2 Data availability statement

The datasets and demographic information including sex, age, histology, survival rate, and outcome were obtained from the The Cancer Genome Atlas (TCGA) database (https://portal.gdc.cancer.gov/). The analysis of data was conducted utilizing the limma package. The adjusted fold change is greater than 1.5. The cut‐offs for screening differentially expressed genes (DEGs) were established as *p* < 0.05. Kaplan–Meier graphs were created to demonstrate the relationship between the overall survival of patients, which was then evaluated using a log‐rank test.

### 2.3 Cell culture

A549 and 95D cells (Type Culture Collection of the Chinese Academy of Sciences cell bank, Shanghai, China) were cultured in DMEM (Thermo Fisher Scientific, MA, USA) containing 10% FBS (Gibco, NY, USA).

### 2.4 Viability analyses

Cells were harvested using trypsin, counted with a Countess Automated Cell Counter, and added to a 96‐well plate (2000/ well in 200 μL). Viability was then assessed by adding AlamarBlue based on provided instructions.

### 2.5 qRT‐PCR

Cellular RNA was assessed via RNeasy mini kit (TIANGEN, CHINA), and RNA concentrations were measured before cDNA was prepared. All qRT‐PCR reactions were performed as protocol. Thermocycler settings were as follows: 94°C for 2 min; 40 cycles of 94°C (30 s), 60°C(30 s), and 72°C (40 s). GAPDH served as a normalization control.

Primers were as follows:

GJB2‐F: CGGTTAAAAGGCGCCACGG.

GJB2‐R:ACGGTGAGCCAGATCTTTCC;

GJB3‐F: TGAGAAGTGCTCCCAAGCAG.

GJB3‐R:CAAGTGATGACCTGGGGGAC;

NFE2L1‐R: TGACATTCTGATTGATGGGAGTGT.

GAPDH‐F: CGGAGTCAACGGATTTGGTCGTAT.

GAPDH‐R: AGCCTTCTCCATGGTGGTGAAGAC.

### 2.6 Western blotting

Protein levels in the extracts were measured using a Bradford assay after lysing cells with chilled RIPA buffer that included protease and phosphatase inhibitors. Afterward, the samples were divided within NuPAGE Novex 4–12% Bis‐Tris Protein gel and moved onto PVDF membranes. The membranes were then blocked for half an hour and probed with primary antibodies overnight. To visualize protein bands, a Western Blot System called ECL (manufactured by GE Healthcare Life Sciences, located in the United States) was utilized. The FUJI Multi Gauge V3.0 analysis program was utilized to measure the non‐saturated bands. The primary antibodies used were anti‐Connexin26 (Rabbit, ab65969, Abcam), anti‐Connexin31 (Rabbit, ab236620, Abcam), anti‐NFE2L1 (Rabbit, ab137572, Abcam), and anti‐β‐actin (A5316, Mouse, Sigma Aldrich).

### 2.7 Immunofluorescence

NSCLC cells were fixed with 4% paraformaldehyde, permeabilized with 0.4% Triton X‐100, and washed with PBS before incubation for 2 h in 5% BSA at 25°C to facilitate blocking. Samples were then probed overnight at 4°C with anti‐NFE2L1 (ab238154, Abcam, 1: 200) in BB, followed by washing with PBS and staining at room temperature for 1 h with AF488‐conjugated goat‐anti‐mouse IgG (1: 100). The epifluorescence Zeiss Axioskop and a Zeiss (Achroplan 40/.75 W objective) were used to capture uncompressed 24‐bit TIFF files. A cooled (−12°C) single CCD color digital camera (Pursuit, Diagnostic Instruments) driven by the freely available SPOT version 4.7 software was utilized for this purpose. To detect AF488 (Chroma Technology), a filter set 41,001 with high IQ was employed. To determine the ratios of nuclear/cytoplasmic, the measurement of average fluorescence intensity was conducted for NFE2L1 localization in both the nucleus and the immediate surrounding area (cytoplasm). Zeiss analysis imaging software, which is freely available, was utilized for conducting all Image analysis. Eighty percent of the population was represented by the chosen cells, which were shown in the relevant figures.

### 2.8 Immunohistochemistry

Tissue sections were deparaffinized using xylene, followed by rehydration with an ethanol gradient and treatment with 3% H_2_O_2_ for 5 min. Afterward, the samples were washed with dH2O and exposed to Tris‐EDTA buffer (pH 9.0) for 35 min to aid in the retrieval of antigens. This was followed by two 5‐min rinses with TBST (Dako). After being blocked for 1 hour using blocking buffer (Dako), the samples were washed with TBST and then probed overnight at 4°C with either anti‐Connexin26 (ab65969, Abcam) or anti‐Connexin31 ab236620, Abcam. Afterward, the samples were probed with an HRP‐conjugated secondary antibody for 1 h at ambient temperature, followed by the detection of protein staining using a DAB substrate kit. Nuclei were counterstained with either hematoxylin or Hoechst. Besides, the primary antibody was omitted when preparing negative control samples. Three pathologists examined the immune‐stained sections using a light microscope to evaluate immune‐positive cells. They scored the sections in 10 randomly selected ×20 power fields. Staining intensity was assessed on a scale of 0 (absence of staining), 1 (low staining), 2 (moderate staining), and 3 (high staining). The scoring for the percentage of positive cells was as follows: 1, less than 25%; 2, between 26 and 50%; 3, between 51 and 75%; and 4, greater than 76%. The sum of these two scores was calculated. Each tissue sample was classified into four groups based on the total scores: Group 0 represented samples with staining in less than or equal to 5% of cells, Groups 1–3 indicated weak expression, Groups 4–5 indicated moderate expression, and Groups 6–7 indicated strong expression. In the end, we conducted a statistical comparison between the quantities of cells exhibiting low‐to‐weak expression and those displaying moderate‐to‐strong expression.

### 2.9 Luciferase reporter assays

NSCLC cells were transduced using either control lentiviral particles or the NFE2L1 3'UTR‐Lenti‐reporter‐Luciferase lentivirus (pLenti‐UTR‐Luc, MV‐h06936, ABM Inc.). Foll owing 2 weeks, luciferase activity was measured with the Bright‐Glo reagent (Promega, WI, USA) and a Victor3 counter (Perkin Elmer, MA, USA).

### 2.10 Interference transfection

The SiRNA was provided by Shanghai Jikai Company, used for gene silencing. Knockdown sequences were as follows: si‐NFE2L1–3 (3’‐AAUUAAAAAUAAAAUCUACAA‐5′). Transfection efficiency was detected by qRT‐PCR and western blotting after 48 h.

### 2.11 Statistical analysis

Data are means ± SD and were compared using two‐tailed *t*‐tests or one‐way ANOVAs with Dunnett's post‐test, as appropriate. Kaplan–Meier curves and log‐rank tests were used to compare survival outcomes. SPSS 22.0 was used for all statistical testing unless otherwise indicated. *p* < 0.05 was the significance threshold.

## 3 RESULTS

### 3.1 GJB upregulation in NSCLC is correlated with survival and prognosis

**FIGURE 1:**
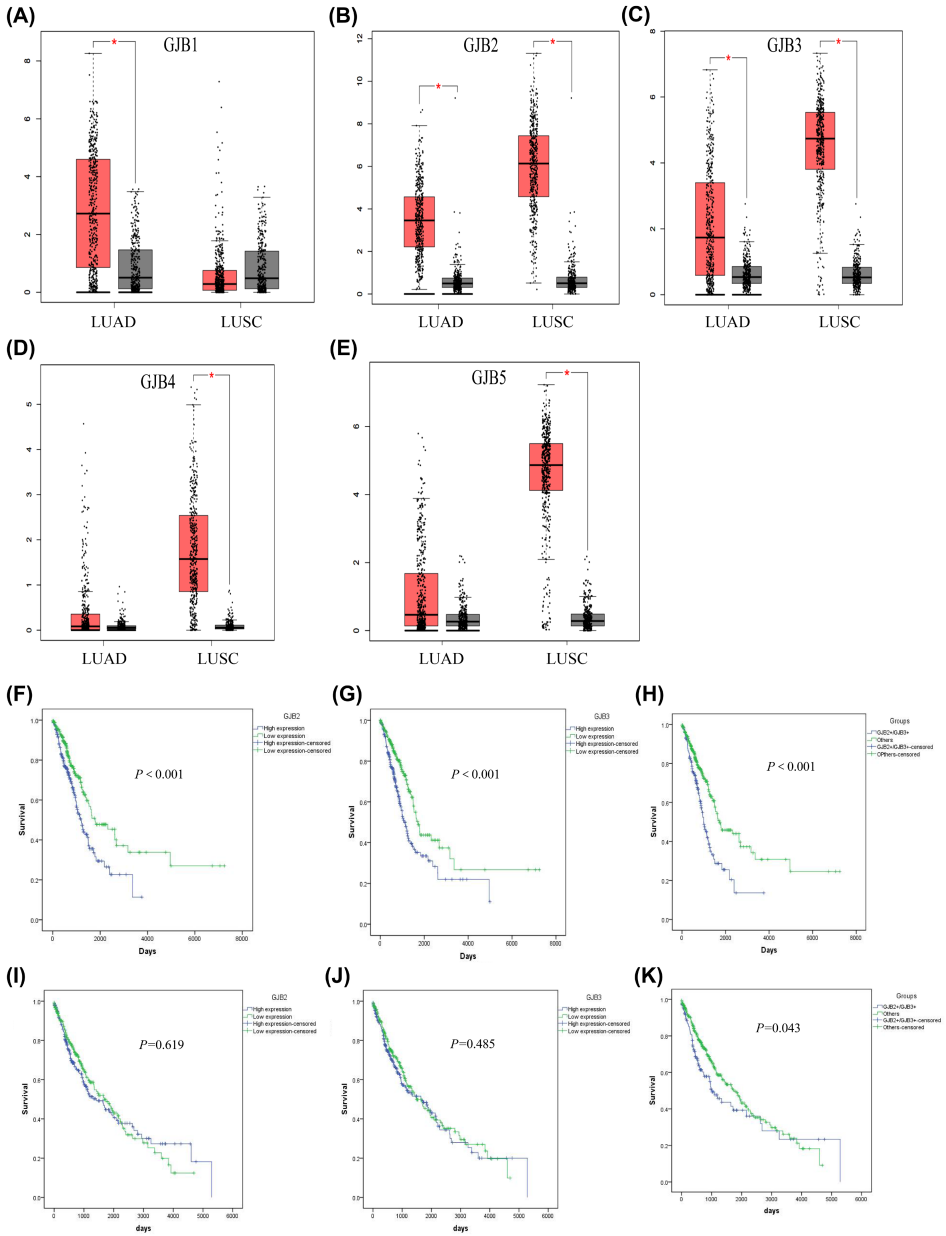
mRNA expression of GJBs and GJB2 and GJB3 survival analysis in the TCGA database. The mRNA expression level of GJB1 (A), GJB2 (B), GJB3 (C), GJB4 (D), and GJB5 (E) in LUAD and LUSC compared with normal lung tissue in the TCGA database; (F) K‐M OS curves based on the expression levels of GJB2 in LUAD; (G) K‐M OS curves based on the expression levels of GJB3 in LUAD; (H) K‐M OS curves based on both high expression levels GJB2 and GJB3 and other cohorts in LUAD; (I) K‐M OS curves based on the expression levels of GJB2 in LUSC; (J) K‐M OS curves based on the expression levels of GJB3 in LUSC; (K) K‐M OS curves based on both high expression levels GJB2 and GJB3 and other cohorts in LUSC, *p<0.05.

We evaluated the expression of GJBs in lung cancer patients through the TCGA and GTEx databases, revealing these genes were significantly upregulated in NSCLC samples compared to normal tissues. Of the five analyzed GJBs, we found both GJB2 (connexin 26 [Cx26]) and GJB3 (connexin 31 [Cx31]) were significantly upregulated in LUAD and LUSC (Figure 1A–E), so we opted to study these two genes in subsequent experiments. Individually, we found that GJB2 and GJB3 expression levels were only associated with LUAD patient outcomes in the TCGA database, whereas high expression levels of both GJB2 and GJB3 were significantly associated with patient prognosis in both LUAD and LUSC (Figure 1F–K). As such, GJB2 and GJB3 represented promising prognostic biomarkers in NSCLC (*p* < 0.05).

**FIGURE 2:**
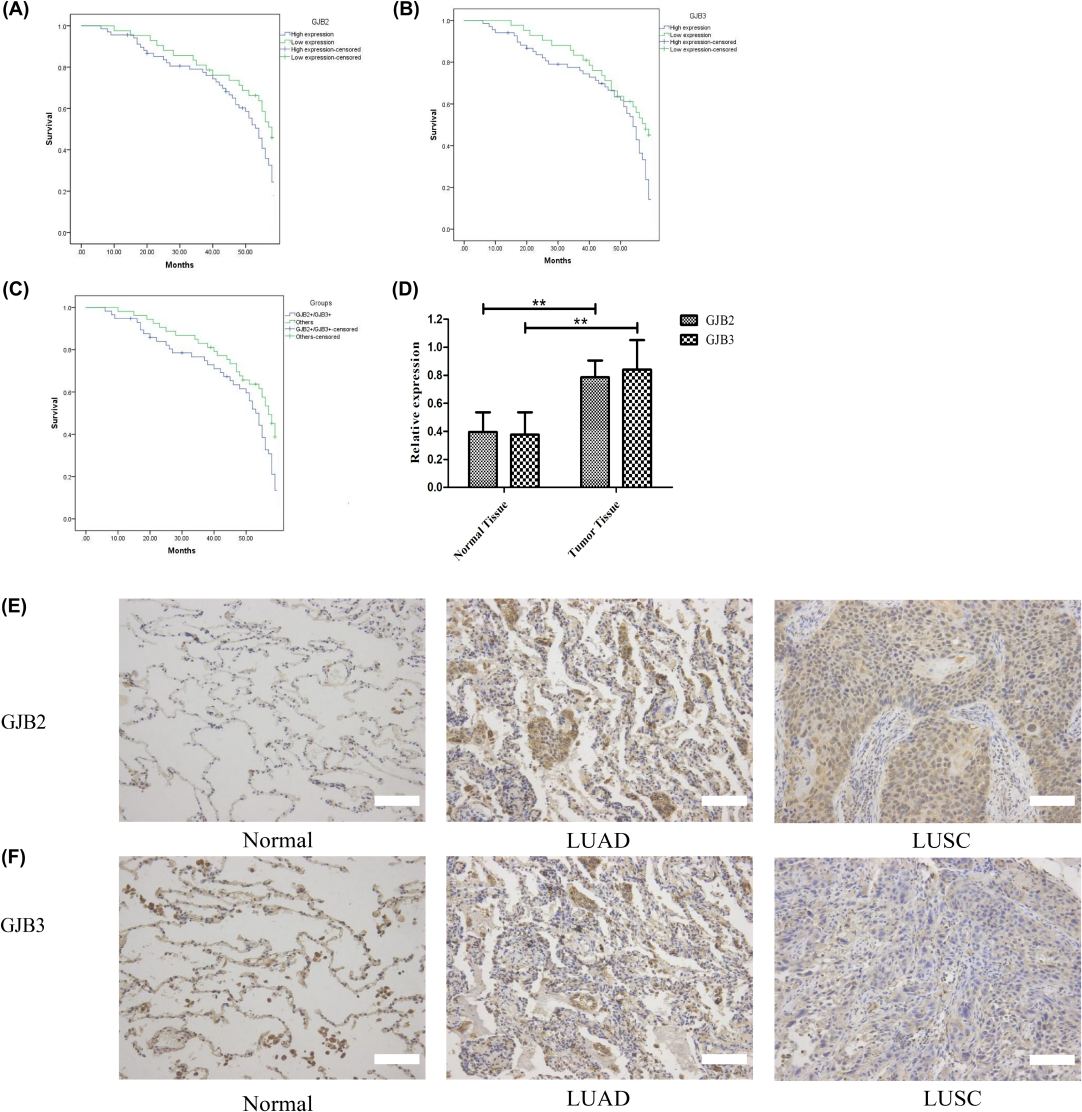
Clinical specimen data analysis. Over‐survival curve analyses curves based on the expression levels of GJB2(A), GJB3(B), and both high expression (GJB2 and GJB3) cohorts compared with other cohorts(C) of NSCLC patients using the log‐rank test. (D) mRNA expression level in NSCLC tissue and paired adjacent non‐tumors tissues. Immunohistochemistry of GJB2 (E) in normal lung tissue, LUAD, and LUSC. Immunohistochemistry of GJB3 (F) in normal lung tissue, LUAD, and LUSC (the scale bars of E F pictures are 100 μm).

Next, we evaluated the relationships between GJB expression and NSCLC patient prognosis. All 116 patients had follow‐up data available through 2020 and were stratified according to their GJB2/GJB3 expression. Patients with both GJB2 and GJB3 positive have a low survival rate, consistent with the TCGA datasets (Figure 2A–C). We conducted immunohistochemical staining of NSCLC tissue samples, revealing significant increases in GJB2 and GJB3 levels in tumor tissue samples compared to the control tissues (Figure 2D–F). GJB2 was over‐expressed in 65.5% (72/110) of tumor tissues and 51.8% (57/110) of normal tissues, while GJB3 was over‐expressed in 61.8% (68/110) and 48.2% (53/110) of the tumor and normal tissues. As such, the average expression of these two connexins was significantly increased in NSCLC tumor tissues relative to normal tissue samples.

Patient clinicopathological findings are summarized in Table 1. We found that increased GJB2 expression was linked to SCC histological classification (*p* = 0.045) and TNM stage (*p* = 0.022), while GJB3 expression was linked to larger tumor size (*p* = 0.045) and increased lymph node metastasis (*p* = 0.012) (Table 1).

**TABLE 1:** Association of GJB2 and GJB3 expressions with clinicopathological features for NSCLC patients.

Clinicopathologic	*N* (%)	GJB2		*p*	GJB3		*p*
Parameters		Positive	Negative		Positive	Negative	
Age							
<60 y	53 (48.1)	33	20	0.497	31	22	0.488
≥60 y	57 (51.9)	39	18		37	20	
Gender							
Male	60 (54.5)	38	22	0.353	34	26	0.223
Female	50 (45.5)	34	16		34	16	
Smoking history							
Yes	47 (42.7)	35	12	0.086	31	16	0.440
No	63 (57.3)	37	26		37	26	
Tumor size							
<3 cm	36 (32.7)	20	16	0.133	16	20	0.045*
3–7 cm	44 (56.9)	34	12		31	13	
≥7 cm	30 (27.6)	24	8		20	10	
Histological classification							
SCC	69 (62.7)	50	19	0.045*	47	22	0.078
AC	41 (37.3)	22	19		21	20	
Histological grade							
Well/moderate	44 (40.0)	26	18	0.252	28	16	0.749
Poor	66 (60.0)	46	20		40	26	
TNM stage							
I + II	53 (48.2)	29	24	0.022*	30	23	0.278
III+ IV	57 (51.8)	43	14		38	19	
Lymph node metastasis							
Positive	56 (50.9)	41	15	0.180	41	15	0.012*
Negative	54 (49.1)	37	23		27	27	
Distant metastasis							
Positive	47 (42.7)	35	12	0.086	32	15	0.243
Negative	63 (57.3)	37	26		36	27	

* *p <* 0.05.

### 3.2 Metformin suppresses GJB expression in NSCLC cells

MET is a hypoglycemic drug that has been shown to suppress NSCLC tumor growth, with one prior study having demonstrated that MET may suppress intercellular communication within the TME. We therefore sought to determine whether MET altered GJB expression levels in NSCLC.

**FIGURE 3:**
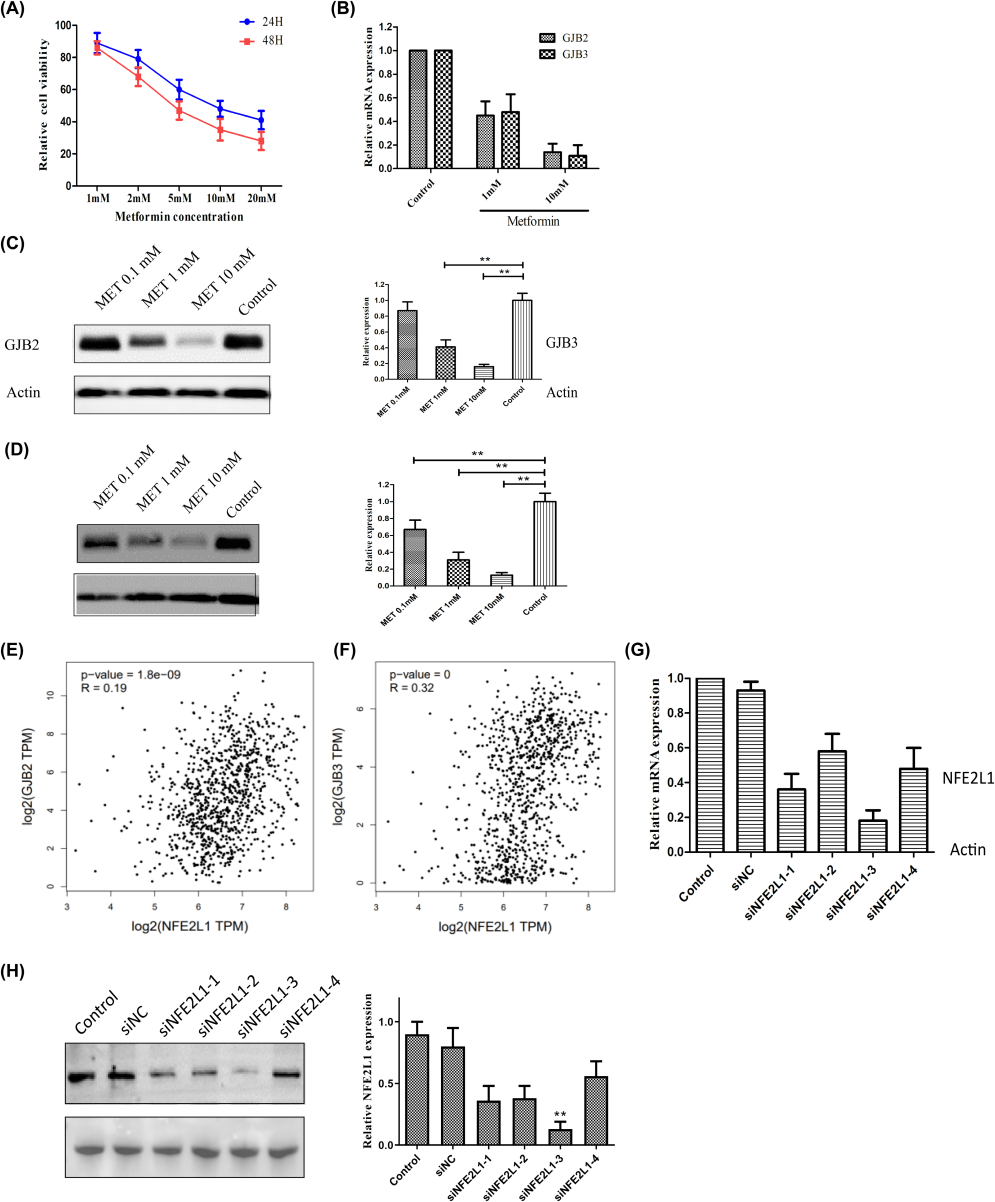
MET influenced GJB2 and GJB3 expression in the A549 cell line. (A) Viability of A549 after exposure with different concentrations of MET after 24H or 48H. (B) mRNA level of GJB2 and GJB3 after exposure with different concentrations of MET after 24H in the A549 cell line. The protein level of connexin 26 (C) and connexin 31 (D) after exposure with different concentrations of MET after 24H in the A549 cell line (the above protein images were all repeated for three experiments). The coregulation analysis between NFE2L1 and GJB2 (E) or GJB3 (F) in NSCLC tissue is based on TCGA data. (G) mRNA level of NFE2L1 after being infected with different siRNA sequences. (H) The protein level of NFE2L1 after being infected with different siRNA sequences.

We began by treating A549 LUAD cells with a range of MET doses (1–20 mM) for 24 or 48 h, after which we evaluated their proliferation via the MTT assay. This analysis revealed a dose‐dependent inhibition of tumor cell proliferation (Figure 3A). We then evaluated GJB2 and GJB3 mRNA and protein levels in A549 cells treated with MET (0.1, 1, or 10 mM), revealing that these GJBs were significantly downregulated in response to MET treatment in a dose‐dependent fashion (Figure 3B–D).

### 3.3 NFE2L1 is involved in the regulation of GJBs in NSCLC

We have previously identified NFE2L2 as a prognostic biomarker of NSCLC.^28^, ^29^, ^30^, ^31^, ^32^, ^33^ As NFE2L1 is a paralog of NFE2L2, it may be similarly related to NSCLC patient outcomes. As such, we examined the relationship between the expression of NFE2L1 and GJB2/3 in the TCGA database, revealing the expression of this transcription factor to be strongly correlated with the expression of both of these GJBs (GJB2: *p* = 1.8e‐09; GJB3: *p* = 0) (Figure 3E,F). We then knocked down NFE2L1 using a siRNA approach using siRNAs from Jikai Co. (Shanghai, China), with si‐NFE2L1‐3 (3’‐AAUUAAAAAUAAAAUCUACAA‐5′) being used for these experiments as it exhibited maximal knockdown efficiency in A549 cells (Figure 3G,H).

**FIGURE 4:**
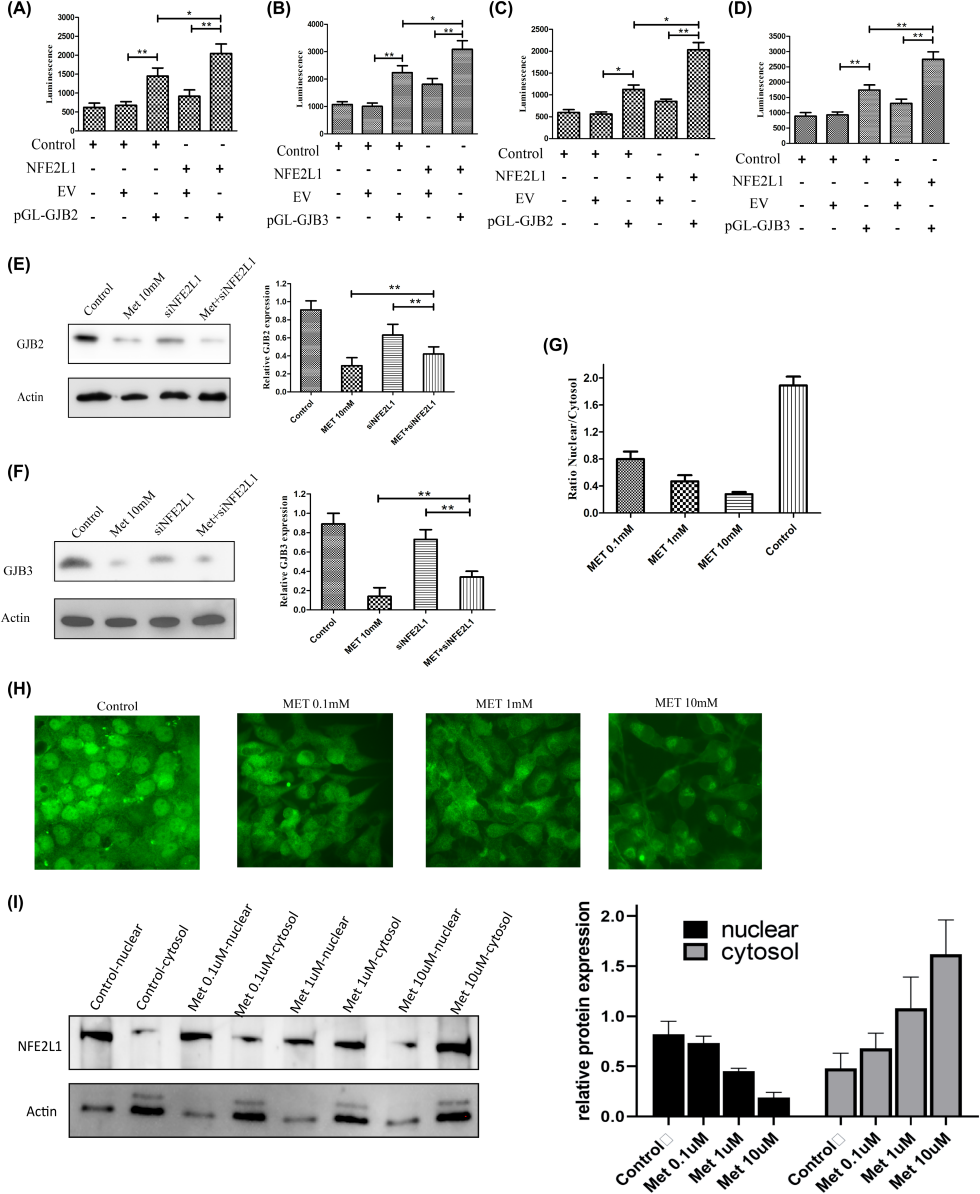
The mechanism of NFE2L1 regulating GJB2 and GJB3 expression under MET exposure. (A–D) Luciferase activity assay showed an increase in transcription activity of GJB2 and GJB3 promoter in NFE2L1 over‐expressed A549 and 95D cells (*n* = 3, *p < 0.05, **p < 0.01, with one‐way ANOVA followed by Dunnett's post‐test). Western blot for GJB2 (E) and GJB3 (F) expression in A549 cells infected with control or siNFE2L1 lentivirus, with or without MET exposure. β‐actin served as a control (the above protein images were all repeated for three experiments). (G) qRT‐PCR for NFE2L1 expression in A549 cells infected with control or siNFE2L1 lentivirus (*n* = 3, *p < 0.05, **p < 0.01, with *t*‐test). (H) Immunofluorescence analysis of NFE2L1 location in A549 cells after exposure with different concentrations of MET (the scale bar of H picture is 10 μm). (I) Protein immunoblotting experiments demonstrated that metformin treatment inhibited the nuclear entry of NFE2L1 and its retention in the cytoplasm.

To more fully explore the functional interaction between NFE2L1 and GJBs in LUAD, we next transfected 95D cells with Empty vector (EV) or GJB2/GJB3 promoters (pGL‐GJB2 and pGL‐GJB3) and evaluated the luciferase activity driven by these promoters in cells over‐expressed NFE2L1 (Figure 4A–D). This analysis revealed that NFE2L1 upregulation induced both GJB2 and GJB3 promoter activation. Together, these findings thus suggested that NFE2L1‐dependent GJB2/GJB3 expression may play a role in LUAD progression.

### 3.4 Metformin suppresses the expression of GJBs in LUAD by suppressing the activation of the NFE2L1 pathway

Western blotting revealed that MET‐mediated inhibition of GJB expression was decreased in cells in which NFE2L1 had been knocked down relative to control cells, suggesting that MET plays an important role in regulating GJB expression (Figure 4E,F).

To determine whether MET influences GJB2/GJB3 expression by modulating NFE2L1 pathway activation in NSCLC, we next measured NFE2L1 expression following MET treatment in A549 cells. Interestingly, NFE2L1 expression was unaffected by MET treatment in these cells. However, immunofluorescent staining assays revealed that NFE2L1 localized to the nucleus in untreated cells, whereas it primarily localized to the cytoplasm in MET‐treated cells (Figure 4G). This change was dose‐dependent and was confirmed by quantifying the NFE2L1 nuclear/cytoplasmic ratio in untreated and treated cells (Figure 4H). Protein immunoblotting experiments demonstrated that metformin treatment inhibited the nuclear entry of NFE2L1 and its retention in the cytoplasm (Figure 4I).

## 4 DISCUSSION

Communication between cells is an integral component of the TME, enhancing the malignant behavior of tumor cells. NSCLC is a deadly form of cancer that exhibits extensive intercellular communication that constrains the efficacy of conventional radiotherapy and chemotherapy based treatment efforts. Cells can communicate through both the secretion of a diverse range of factors and through direct cell–cell contact. Recent evidence suggests that a range of key proteins are involved in lung cancer progression, including connexins, pannexins, and tight junction proteins.^13^, ^14^, ^15^

While many studies have explored the role of GJA1 (connexin 43) in NSCLC, the role of GJBs in lung cancer remains poorly understood. Under hypoxic conditions, Cx26 internalization has been shown to drive cancer cell proliferation and epithelial‐mesenchymal transition (EMT) via P53/MDM2 signaling pathway activation.^34^ Gefitinib‐resistant (GR) NSCLC cells have also been shown to exhibit Cx26 upregulation, and knocking down this connexin reverses GR and EMT progression in both HCC827‐GR and PC9‐GR cells through a mechanism dependent upon PI3K/Akt signaling.^35^ In papillary thyroid cancer, Cx26 has also been linked to larger tumor size and lymph node metastases,^36^ while in colorectal cancer it has been associated with an increase in lung metastasis and a decrease in lung metastasis‐free survival, whereas it was unrelated to liver or lymph node metastasis. Cx26‐positive lung cancer cells are more invasive and metastatic, suggesting that this connexin may be an independent predictor of patient prognosis that is expressed on the surface of SCC cells facing the tumor stroma or capsule.^23^

There have been few studies of Cx31 in the context of cancer today. One study of hearing loss suggested that Cx31 mutations may influence GJ formation through mechanisms associated with Cx26. Cx31 has also been detected between basal cells in the airway, and it is highly expressed in long‐term cultures of well‐polarized airway epithelial cells, suggesting it may play a critical role in the development of airway tissues.^37^

To explore the functions of GJBs in NSCLC, we analyzed the expression of these genes in NSCLC patient tumors and paracancerous tissues from the TCGA database. Of the five GJBs, we found that only GJB2 and GJB3 were differentially expressed in LUAD and LUSC tumors relative to normal tissues, while the expression of GJB4 and GJB5 was not detectable. We then conducted immunohistochemical staining for Cx26 and Cx31, allowing us to confirm that high expression of these GJBs was associated with a poorer prognosis in both LUAD and LUSC patients. Lung cancer is most commonly attributable to smoking, which is associated with 80% of cases.^38^ While Cx26 trended toward upregulation in tobacco smokers, this increase was not significant. Cx31 was expressed at higher levels in larger tumors. As such, different connexins exhibit different degrees of prognostic relevance in the context of tumor progression.

As a metabolism‐regulating medication, MET has been shown to decrease cancer risk and improve the survival of lung cancer patients. Indeed, NSCLC patients harboring LKB1^39^ or p53^40^ mutations are more likely to benefit from the antitumor properties of MET. In one study, Met was shown to improve the survival of lung cancer patients with type 2 diabetes mellitus (T2DM) undergoing EGFR‐TKI therapy.^41^

In one recent study, the OS of NSCLC patients with T2DM treated with MET was increased by 20% relative to patients not taking MET.^42^ MET has also been shown to improve progression‐free survival in NSCLC patients with stage I and II disease after pulmonary resection.^43^ Given that GJB2/GJB3 are related to the prognosis of NSCLC, we evaluated the impact of Cx26 and Cx31 on A549 cells. We discovered that MET treatment was associated with marked GJB downregulation. Given that MET has been repeatedly been shown to suppress NSCLC tumor malignancy, we next explored the mechanisms whereby MET influences connexin expression.

We have previously identified NFE2L2 as a prognostic factor in the context of NSCLC.^44^, ^45^ In this study, we therefore explored the relevance of the paralogous NFE2L1 in NSCLC, given that it has not previously been studied in this cancer type. There is evidence that NFE2L2 is a key regulator of inflammation, metabolism, oxidative stress, and proteostasis.^46^ Treating human cells with TGF‐β leads to NFE2L1 upregulation,^47^ while NFE2L1 knockdown can lead to dysregulated lipid metabolism and altered fatty acid composition.^48^, ^49^ Waku et.al^50^ found that NFE2L1 maintains basal proteasomal activity in CRC cells. NFE2L1 knockdown was also shown to promote insulinoma cell proliferation and invasion in vitro and chemoresistance in a murine allograft transplantation model, highlighting NFE2L1 as a potential tumor suppressor.^51^ In hepatoma cells, NFE2L1 has also been shown to function as a mitochondrial retrograde gene that drives invasion and hepatic progression.^52^ In A549 and PC9 NSCLC cells, NFE2L1 overexpression has also been shown to drive LUAD progression via regulating the WNT pathway.^53^ The NFE2L1 gene is also associated with obesity,^54^ and NFE2L2 overexpression results in weight loss and protection against diet‐induced obesity in transgenic mice.

Overall, these data led us to hypothesize that NFE2L1 may function as an intracellular receptor that regulates cell–cell communication in response to MET treatment. Consistent with such a model, we found that the overexpression of NFE2L1 promoted GJB2 and GJB3 promoter activation in A549 cells, whereas knocking down NFE2L1 partially reversed the MET‐mediated suppression of GJB expression.

Communication occurs not only between tumor cells but also between tumor cells and immune or stromal cells. As the present study solely evaluated the role of NFE2L1 as a regulator of GJB expression in tumor cells, further in vivo work will be required to understand the functional role of this signaling pathway more fully in the context of a complex TME. In one recent study, connexins were shown to be differentially expressed in primary and metastatic tumors, underscoring a potential direction for future work. Additional studies regarding the role of connexins in NSCLC may lead to the development of novel treatments for this deadly cancer type.

## AUTHOR CONTRIBUTIONS

**Shuo Yu:** Writing – original draft (equal). **Hui Ren:** Writing – original draft (equal). **Tingting Liu:** Writing – original draft (equal). **Xiaoyan Han:** Data curation (equal). **Hui Guo:** Data curation (equal). **Qian Ning:** Methodology (equal). **Yang Li:** Resources (equal). **Hong Zhou:** Methodology (equal). **Mingwei Chen:** Writing – review and editing (equal). **Tinghua Hu:** Writing – review and editing (equal).

## FUNDING INFORMATION

This study was supported by the Exploration and Innovation Project of First Affiliated Hospital of Xi'an Jiaotong University (No. 2019ZYTS‐05); Shaanxi Provincial Key Research and Development Program (No. 2021KW‐51); Natural Science Basic Research Plan in Shaanxi Province of China (No. 2022JQ‐791); and Special Fund for Personnel Training of Second Affiliated Hospital of Xi'an Jiaotong University (No. RC(XM)202007).

## CONFLICT OF INTEREST STATEMENT

We declare there are not relative competing interests about our manuscript.

## ETHICS STATEMENT

The Ethics Committee of Clinical Research of Xi'an Jiao tong University approved this study, which aligned with the Declaration of Helsinki and local guidelines. All patients provided written informed consent.
